# Insights from the Infection Cycle of VSV-ΔG-Spike Virus

**DOI:** 10.3390/v14122828

**Published:** 2022-12-19

**Authors:** Elad Milrot, Shlomi Lazar, Ofir Schuster, Efi Makdasi, Shlomo Shmaya, Yfat Yahalom-Ronen, Hadas Tamir, Orly Laskar

**Affiliations:** 1 Department of Infectious Diseases, Israel Institute of Biological Research (IIBR), Ness-Ziona 74100, Israel; 2 Department of Pharmacology, Israel Institute of Biological Research (IIBR), Ness-Ziona 74100, Israel

**Keywords:** viral factories, vesicular stomatitis virus, spike protein, transmission electron microscopy, electron tomography, RNA virus

## Abstract

Fundamental key processes in viral infection cycles generally occur in distinct cellular sites where both viral and host factors accumulate and interact. These sites are usually termed viral replication organelles, or viral factories (VF). The generation of VF is accompanied by the synthesis of viral proteins and genomes and involves the reorganization of cellular structure. Recently, rVSV-ΔG-spike (VSV-S), a recombinant VSV expressing the SARS-CoV-2 spike protein, was developed as a vaccine candidate against SARS-CoV-2. By combining transmission electron microscopy (TEM) tomography studies and immuno-labeling techniques, we investigated the infection cycle of VSV-S in Vero E6 cells. RT-real-time-PCR results show that viral RNA synthesis occurs 3–4 h post infection (PI), and accumulates as the infection proceeds. By 10–24 h PI, TEM electron tomography results show that VSV-S generates VF in multi-lamellar bodies located in the cytoplasm. The VF consists of virus particles with various morphologies. We demonstrate that VSV-S infection is associated with accumulation of cytoplasmatic viral proteins co-localized with dsRNA (marker for RNA replication) but not with ER membranes. Newly formed virus particles released from the multi-lamellar bodies containing VF, concentrate in a vacuole membrane, and the infection ends with the budding of particles after the fusion of the vacuole membrane with the plasma membrane. In summary, the current study describes detailed 3D imaging of key processes during the VSV-S infection cycle.

## 1. Introduction

Viruses are obligate intracellular infectious parasites that rely on host machineries for replication and assembly. Accordingly, viruses manipulate cellular organization, metabolism and biochemical pathways to support effective reproduction [1,2]. Key processes in the infectious cycle, such as transcription, genome replication and virus assembly, are usually carried out in highly compound and dynamic structures termed viral factories (VF) [2,3,4,5]. Viral factories usually consist of host organelles and cytoskeleton elements supporting virus assembly and transport. Mitochondria, ER or Golgi membrane networks are usually seen adjacent to VF and assumed to play a role in energy production, scaffolding replication complexes and production of viral proteins, all necessary for effective virus propagation [6,7,8]. Occasionally, morphogenesis of (+) RNA viruses requires several maturation steps that occur in distinct membrane structures, involving several host membranous organelles [4]. Togavirus VF are organized around modified endosomal and lysosomal membranes, forming cytopathic vacuoles, unique to these virus’ replication [7]. Flavivirus infection induces rearrangement of cytoplasmic membranes in the perinuclear region, where a factory-like structure is formed. Distinct membrane morphologies, such as membrane spherules, most probably originating from ER, were observed in infected cells. Members of the *Coronaviridae* (SARS-CoV) generate a unique reticulovesicular network of modified endoplasmic reticulum that integrates convoluted membranes with numerous interconnected double membrane vesicles, in which both viral replicase subunits and dsRNA are associated [9]. (−) RNA viruses such as Bunyaviruses harness the Golgi complex to support viral RNA replication and virus particle assembly [10]. (−) RNA viruses, such as members from *Rhabdoviridae* family, Vesicular Stomatitis Virus (VSV) and rabies, were investigated throughout the years. Rabies viral genome replication and gene transcription occur in distinct cytoplasmic inclusion bodies (IBs) consisting of viral proteins and nucleic acids [11,12]. It was shown that the IBs (also termed “Negri bodies”) have granular and filamentous materials consisting of viral nucleoproteins and newly formed viruses [11,13]. The IBs have characteristics resembling that of liquid organelles, i.e., they are spherical, can fuse and form large compartments, and disappear after hypotonic shock [14]. Similarly, VSV reproduction is associated with the formation of compartments with liquid-like properties [15]. VSV mRNA is synthesized throughout the cell cytoplasm, however, RNA synthesis occurs at distinct sites in the cytoplasm after viral protein translation [16]. Similar to rabies, the sites of VSV RNA synthesis contain viral proteins but exclude host’s membranes [17]. Although IBs are devoid of host membranes at early stages of formation, ER membranes were seen adjacent to IBs at late stages of infection or even completely surrounding them [11].

From the various RNA viruses’ VF (from a variety of families) investigated so far, each virus has its own distinct strategy for generation of VF in a specific cellular organelle or set thereof [4].

Transmission Electron Microscopy (TEM) is a fundamental tool used for imaging virus particles as well as studying virus morphogenesis in infected cells or tissues [8,18]. Advanced correlative electron microscopy approaches combined with high resolution fluorescence microscopy techniques are applied for imaging viruses in the context of the host environment. These powerful tools enable the locating of specific viral proteins inside infected cells at high precision. However, these techniques are laborious and require expensive microscopes and highly trained personnel [19,20].

Although TEM is useful in virus research, analysis of thin sections of infected cells merely provides 2D projection snapshots of specific time points, with cellular organization in space being lost. One of the techniques used to overcome this issue is Electron Tomography (ET) [18]. TEM ET technique uses a set of 2D images, recorded at different specimen tilt angles with respect to the primary beam, generating a 3D tomogram. Typically, the specimen is tilted up to ~67° in small tilt increments (~1.5°), with an image recorded at each tilt angle. After aligning the set of 2D images, special algorithms are used to generate a 3D tomogram [21]. TEM ET is widely used to investigate virus entry into cells, assembly and cell egress, making it a powerful tool which enables the understanding of the mechanistic aspects of virus infection [12,16].

The recent Coronavirus pandemic has resulted in significant global health and economical burdens. Despite vaccination programs, the emergence of new variants results in ongoing infections. Therefore, clinical trials of new vaccine candidates remain relevant. VSV is a (−) RNA virus belonging to the *Rhabdoviridae* family that is characterized by a “bullet”-like morphology. The virus has a lipid envelope (decorated with G proteins), enclosing a nucleocapsid composed of RNA and nucleoprotein N and an associated matrix formed by M proteins [22,23,24]. The virus has been used as a vaccine vector for human pathogenic viruses such as Ebola (FDA approved), HIV, SARS-CoV-2 and influenza (under studies) [25,26,27,28]. Recently, rVSV-ΔG-spike (VSV-S), a recombinant VSV that carries the spike protein of SARS-CoV-2 in place of the native G-protein, was developed in our institute as a vaccine candidate against COVID-19 [29]. The current study aims to investigate morphological key stages in the infection cycle of VSV-S by combining TEM electron tomography studies and immuno-labeling techniques. 

## 2. Materials and Methods

### 2.1. Cell Lines

Vero E6 (CRL-1586) cell line was obtained from the American Type Culture Collection (ATCC). Cells were cultured in Dulbecco’s modified Eagle’s medium (DMEM), containing 10% (*v*/*v*) fetal bovine serum (FBS), 1% L-glutamine, 1% (*v*/*v*) non-essential amino acid (NEAA) and 0.5% (*v*/*v*) antibiotics, and incubated at 37 °C under 5% CO_2_ in a humidified incubator. For virus infection of Vero E6 cells, we used DMEM containing 2% FBS and the above supplements (infection medium). All reagents were purchased from Biological industries, Beit Haemek, Israel.

### 2.2. Viruses

In the current study we used VSV-S, a recombinant VSV that carries the spike protein of SARS-CoV-2. The virus was generated in our facility as a vector vaccine candidate against SARS-CoV-2 [29]. The vector is based on wild type VSV (Indiana strain). SARS-CoV-2 (GISAID accession EPI_ISL_406862) was kindly provided by the Bundeswehr Institute of Microbiology, Munich, Germany.

### 2.3. Stains

DAPI stock solution of 1 mg/mL (Sigma, Rehovot, Israel), ER staining kit Red Fluorescence Cytopainter (Cat. 139482, Abcam, Cambridge, MA, USA), Concanavalin A Alexa fluor 594 Conjugate (Cat. 11253, Invitrogen, Molecular probes, Darmstadt, Germany). Stock solutions were prepared according to manufactures protocol. 

### 2.4. Antibodies

#### Generation of Rabbit Serum Antibodies

**Figure 1:**
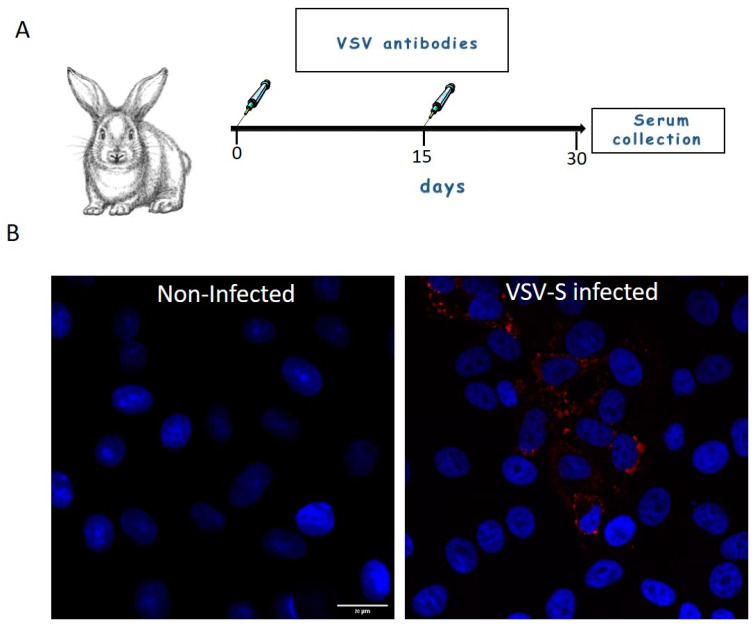
(**A**) Generation of rabbit serum antibodies. Rabbits were immunized with wild type-VSV (day 0) and at day 15 post prime injection, the rabbits were boosted with a similar dose subcutaneously. Serum was collected 15 days post boost. (**B**) Cells were either mock or VSV-S infected and then processed for immuno-florescence microscopy with 1:200 diluted rabbit serum antibodies. VSV proteins (Red) positive signal was visible in cytoplasm of infected cells, whereas in non-infected cells no staining of VSV proteins was seen. Nuclei of the cells are marked with blue color (DAPI). Scale bar is 20 µm.

Rabbits were immunized with wild type-VSV (1.5 × 10^6^ pfu/mL, 1 mL, combined intramuscular and subcutaneous). At day 15 post prime injection, the rabbits were boosted with a similar dose subcutaneously. Serum was collected 15 days post boost (Figure 1A). For immunofluorescence assays, rabbit serum antibodies were diluted 1:200 from stock (Figure 1B). The binding ability of the serum antibodies to VSV proteins was determined by immuno-TEM of negatively stained virus particles (Supplementary Figure S1).

dsRNA antibody clone J2 (Cat. MABE1134, Sigma, Rehovot, Israel) was used in 1:200 dilution from stock. Secondary antibodies Alexa fluor 594 conjugates (Cat. A21207 and A21203, Invitrogen, Molecular probes, Darmstadt, Germany) were used 1:200 from stock solution; FITC conjugated anti rabbit IgG (Cat. F6005, Sigma, Rehovot, Israel) was used 1:100 or 1:200 dilution from stock.

### 2.5. Infection of Vero E6 Cell for RT Real Time PCR

Vero E6 cells at density of 200,000 cells/well were seeded in 24 well plates. After cell adherence, the cells were infected with VSV-S at MOI of 1 (pfu/cell) for 1 h at 4 °C to allow virus adsorption. Then, cells were washed with infection medium and supplemented with fresh infection medium containing 0.15% bicarbonate (Cat.03-040-1B, Biological Industries, Beit Haemek, Israel), and quickly moved to a 37 °C incubator to allow virus internalization. At the desired time point post infection, the soup was discarded and the cells were added with 350 µL of RLT buffer (RNeasy mini kit, Cat.74104, Qiagen, Valencia, CA, USA), after which the cells were scrapped and transferred to Eppendorf tubes for storage at −70 °C until RNA extraction. RNA was extracted according to supplier’s protocol.

### 2.6. Reverse Transcription (RT)-Real Time PCR

RT-Real time PCR was performed using the Sensi FAST^TM^ probelo-ROX one-step kit (Cat. BIO-72001, Bioline, London, UK). The sequence of the primers and probes used for the detection of S (spike) and N (nucleocapsid) genes were published elsewhere [29]. RT-Real time PCR conditions and program were performed according to the manufacturer’s recommendations.

### 2.7. Immunofluorescence Assay of VSV-S Infected Vero E6 Cells

Vero E6 cells at density of 100,000 cells/well were seeded in 8 chamber LabTek slides (Nunc, Naperville, IL, USA) or 250,000 cells/well in 24 well plates with glass cover slips at the bottom. After cell adherence, the cells were infected with VSV-S at MOI of 1 (pfu/cell) for 1 h at 4 °C to allow virus adsorption. Then, cells were washed with infection medium and added with fresh infection medium containing 0.15% bicarbonate, and quickly moved to a 37 °C incubator to allow virus internalization. At the end of infection, the cells were fixed by 4% paraformaldehyde for 30 min at 4 °C and washed with PBS. Perforation of the cells was carried out using TrytonX100 solution for 5–10 min. Blocking was carried out in a solution containing 2% BSA-PBS followed by addition of primary antibody for 1 h at 37 °C. The cells were then washed with PBS and a secondary conjugated antibody was added for 1 h at 37 °C. For ER staining, the cells were stained with the ER staining kit Red Fluorescence Cytopainter at 1:500 dilution, or 100 µgr/mL of Concanavalin A Alexa fluor 594 Conjugate for 30 min, followed by washing with PBS. DAPI counter staining was carried out at 1:10,000 dilution (from 1 mg/mL stock solution) for 10 min at room temperature and then the cells were washed. The cover slips containing cells were mounted on glass slides by using Fluoromount (Cat. F4680, Sigma, Rehovot, Israel) followed by air drying.

### 2.8. Chemical Fixation of Wild Type and VSV-S Infected Vero E6 Cells

Vero E6 cells were infected with wild type VSV or VSV-S at MOI of 1 for 1 h at 4 °C to allow virus adsorption. Then, cells were washed with infection medium and added with fresh infection medium containing 0.15% bicarbonate, and quickly moved to 37 °C incubator to allow virus internalization. After infection, the cells were washed once with PBS, scrapped and transferred to Eppendorf tubes containing 2.5% Glutaraldehyde and 2% Paraformaldehyde, in 0.1 M Cacodylate at pH = 7.4 (Cat. 15960-01, Electron Microscopy Sciences, Hartfield, PA, USA). The cells were then washed 3 times in 0.1 M Cacodylate buffer pH = 7.4 and embedded in 4% of Noble Agar (Cat. A5431, Sigma, Rehovot, Israel). The agar embedded cells were chopped into small pieces, roughly 1 mm, and then post fixed for 1 h in a solution containing 1% (*v*/*v*) of osmium tetra-oxide supplemented by 0.5% (*w*/*v*) of potassium hexa-cyanoferrate and potassium di-chromate. The cells were washed several times in 0.1 M Cacodylate buffer pH = 7.4 and then double distilled water (DDW) followed by staining with 2% of uranyl acetate (Cat. 22404, Electron Microscopy Sciences, Hartfield, PA, USA) for 1 h at room temperature. The cells were washed in DDW and then dehydrated in a series of graded ethanol solutions (30%, 50%, 70%, 96%), twice for each solution for 5 min and then 10 min in dry ethanol. The cells were soaked in 30% epoxy resin (Cat. R1140, Agar Scientific, Stansted, UK), diluted in dry ethanol for several hours and then 50% epoxy resin overnight. The samples were then transferred to pure resin and replaced in pure resin several times to allow efficient infiltration. Polymerization was carried out for 48–72 h at 60 °C. 

After polymerization, the samples were cut to thin (roughly 100 nm) or semi-thick sections (200–280 nm) with a diamond knife using EM UC-7 ultra-microtome (Leica, Microsystems, Wetzlar, Germany). The sections were mounted on 150 mesh carbon coated cupper grids (Cat. CF150-Cu, Electron Microscopy Sciences, Hartfield, PA, USA) used for tomography studies or 300 mesh carbon coated cupper grids (Cat., D1843-F, Ted Pella, Redding, CA, USA) used for thin section TEM analysis and post stained with ready-to-use Uranyless stain and lead citrate (Cat. 22409, 22410, Electron Microscopy Sciences, Hartfield, PA, USA) for seven minutes each stain. Grids were washed in DDW five times after the addition of each stain. The grids were allowed to air dry. 

### 2.9. Immuno-TEM for Determination of Specificity of Rabbit Serum Antibodies

Viruses were fixed by 2% paraformaldehyde prior to immuno-TEM studies. The viruses were incubated for 20 min on 200 mesh carbon coated TEM grids (D1840-F, Ted Pella, Redding, CA, USA). The grids were blocked by 2% BSA-PBS for 20 min followed by an incubation with small drops of blocking solution containing 1% BSA, 0.5% gelatin in PBS for additional 20 min. Anti-VSV serum antibodies were used 1:30 dilution in blocking solution. Incubation with antibodies was carried out for one hour at room temperature. The grids were then washed with a solution of 1% Tween-20 in PBS followed by incubation with a 1:30 diluted secondary antibody conjugated to 10 nm gold particles (Goat anti Rabbit IgG, Cat. G3379, Sigma, Rehovot, Israel). The grids were then washed several times in 1% Tween-20 in PBS followed by several washes in DDW. Staining of grids was carried out with 1% Phosphotungstic acid (PTA). After air-drying, samples were visualized in TEM.

### 2.10. TEM Imaging

Images were recorded with a 200 kV Talos F200C TEM microscope (Thermo Fisher Scientific, Hillsboro, OR, USA) equipped with a CETA camera. The 4 × 4 or 2 × 2 images were acquired by Velox software (Version 2.11, Thermo Fisher Scientific, Hillsboro, OR, USA) or Tecnai T12 TEM (Thermo Fisher Scientific, Hillsboro, OR, USA) operated at 120 kV and equipped with a Gatan ES500W Erlangshen camera. 

### 2.11. TEM-Tomography Analysis of VSV-S Infected Cells

For TEM tomography studies, semi-thick sections (200–280 nm thick) were used. After defining the region of interest (ROI), a tilt series of 4096 × 4096 pixel size images were acquired. Automatic sample tilting, focusing and image shift correction, were performed with “Tomography” software (Version 5.2, Thermo Fisher Scientific, Hillsboro, OR, USA). Double tilt series were acquired at 1.5° increments at an angular range of −67 and +67 degrees with CETA camera. The 3D reconstruction of binned data (binning factor of 2) was computed using weighted back projection or SIRT algorithm by “Inspect 3D” software (Version 4.5, Thermo Fisher Scientific, Hillsboro, OR, USA). Tomogram segmentation and visualization were carried out with “Amira” software (Version 6.5, Thermo Fisher Scientific, Hillsboro, OR, USA).

## 3. Results

### 3.1. Early Morphological Events in the Infection Cycle

**Figure 2:**
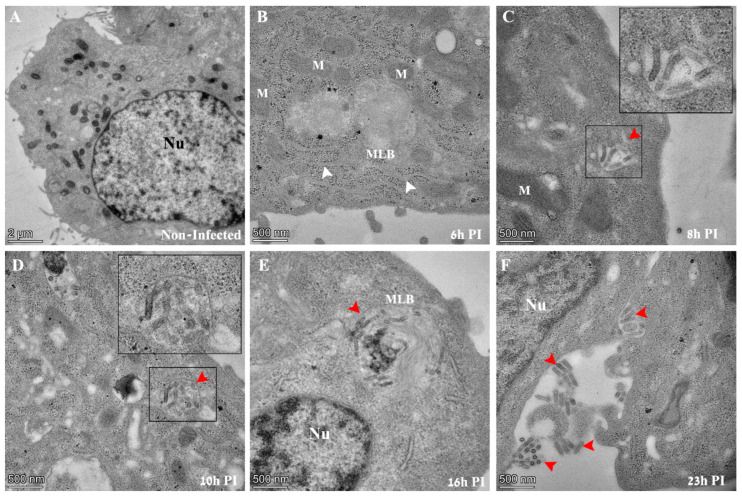
TEM images of thin sections prepared from Vero E6 cells infected with VSV-S. The cells were infected with the virus at an MOI of 1 and then processed by chemical fixation at the indicated time points post infection (PI). (**A**) Non-infected cells show no virus particles in the cytoplasm. The nucleus (Nu) of the cell is visible with surrounding mitochondria. (**B**) Six hours PI, a typical cell exhibits no virus particles in the cytoplasm. Membrane cisternae (white arrows), ribosomes, and multi lamellar bodies (MLBs) are frequently seen. (**C**–**F**) At 8–23 h PI, “bullet like” structures appear in vacuoles (panels (**C**,**D**), red arrows) in cytoplasmic MLBs (panel (**E**), red arrow), or adjacent to the outer leaflet of the plasma membrane (panel (**F**), red arrows). In panels (**C**,**D**), insets show high magnification of delineated areas. M—mitochondria, Nu—nucleus, MLB—multi lamellar bodies.

As mentioned above, the aim of the current study is to investigate key events in the infection process of rVSV-ΔG-spike (VSV-S), a VSV-based vaccine developed in our facility. The virus is produced in Vero E6 cells; consequently, we used these cells for study design. Infection synchronization was carried out by infecting the cells for one hour at 4 °C to allow virus adsorption, after which the cells were washed to remove unbound virus particles. The cells were then immediately transferred to 37 °C to allow synchronized internalization of the absorbed virus particles. The infection was stopped at various time points and the cells were chemically fixed and processed for TEM analysis or immuno-labeling fluorescence microscopy. Figure 2 shows representative TEM images of thin sections of VSV-S infected cells. 

Initial morphological evidence of infection could be seen at 8 h post infection (PI), when typical “bullet like” structures appear inside vacuoles in the cytoplasm of infected cells (Figure 2C, red arrow and inset). As infection extended to later PI time points of 10, 16 and 23 h PI, virus particles were seen in cytoplasmic vacuoles, multi-lamellar bodies (MLBs) or projecting out of the outer leaflet of the plasma membrane (Figure 2D–F, red arrows). The distinction between MLB and vacuoles is based on morphological features (discussed later). No virus particles could be seen at an early time point (6 h PI) or in non-infected cells in TEM sections (Figure 2A,B).

As VSV-S is a pseudo virus, we assessed the morphological effect caused as a consequence of replacing the native G protein that of wild-type VSV, with the spike protein that of VSV-S. Supplementary Figure S2 shows the results from thin section TEM analysis of wild-VSV and VSV-S infected Vero E6 cells. As can be seen, the classical “bullet” shaped VSV particles (white inset in panel A) were seen in cytoplasmic sites devoid of host organelles and ribosomes, and surrounded by host membranes (blue arrows ). In addition, virus particles with various morphologies were frequently seen in cytoplasmic vacuoles (Supplementary Figure S2, panels C,D) [30], similar to VSV-S (Supplementary Figures S2, panels E,F and Figure 2C,D).

**Figure 3:**
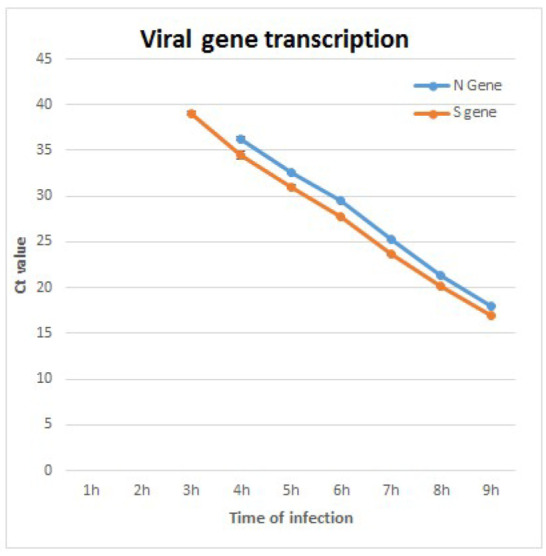
RT real-time PCR analysis results of two viral genes, nucleocapsid (N) and spike (S). Initial detection at 3–4 h post infection (PI) can be seen. As infection progresses, the reduction of Ct values suggests active viral RNA replication. The limit of detection for detection of S gene was 10 pfu/mL, and 100 pfu/mL for N gene. At 1–2 h PI, viral RNA was not detected due to low concentration below the limit of detection.

RT real-time PCR analysis of two viral genes, spike (S) and nucleoprotein (N), detects initiation of transcription at 3–4 h PI, with Ct values lowering over time, signifying an ongoing, progressive process (Figure 3). The combined TEM and RT real-time PCR results imply that viruses observed at 8 h PI and above are de novo synthesized in the cells and not viruses that entered the cells from the beginning of infection.

### 3.2. VSV-S Viral Factories Are Part of a Complex Network Composed of Cellular Membranes and Multi Lamellar Bodies

**Figure 4:**
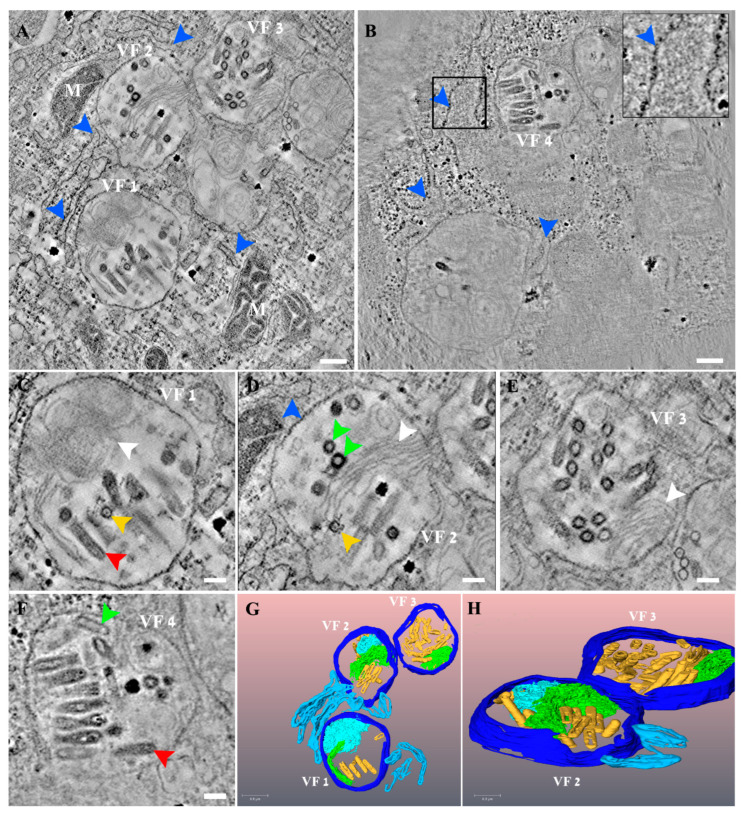
VSV-S viral factories (VF) are part of a complex network composed of cellular membranes and multi lamellar bodies (MLB). (**A**,**B**) Two tomographic slices from two different tomograms of VSV-S infected cells at 16 (**A**) and 23 (**B**) hours post infection (PI). (**C**–**F**) High magnification views of the VF 1–4 shown in panels (**A**,**B**), respectively. VSV-S VF are located in multi lamellar structures with adjacent host membrane cisternae (blue arrows and inset that shows high magnification of delineated area with host ribosomes on a membrane cisterna). The VF consist of empty particles (green arrows) as well as viruses already packed with their genome (red arrows) or other virus morphologies with partial density inside (yellow arrows). White arrows point to “onion like” membrane stacks or linear membrane sheets that are part of the MLB. (**G**,**H**) 3D volume rendering of the tomogram in panel (**A**) showing the MLB “onion like” composition (light blue), stack of membrane sheets (green) and adjacent membrane cisternae (light blue). Various virus morphologies of VSV-S particles depicted in yellow. Scale bars in panels (**A**,**B**) are 200 nm and (**C**–**F**) 100 nm. Thickness of tomographic slices are 8.5 nm (**A**), 6.8 nm (**B**).

In order to investigate the 3D organization of VSV-S factories, we used TEM electron tomography (ET) of VSV-S infected cells. Semi-thick sections of chemically fixed samples were taken at different time points post infection. We focused on 10–24 h PI, as prominent infection of the cells was observed at these time points by thin section TEM analysis. Figure 4A,B show tomographic slices from two different tomograms of VSV-S infected cells, at 16 and 23 h PI. Panels C-F show high magnification views of the respective VF1-VF4 in panels A and B. As shown, VSV-S generates its factories in MLBs in the cells’ cytoplasm. The factories are surrounded by host mitochondria, membrane vesicles and cisternae. Occasionally, ribosomes are seen coating these cisternae (Figure 4B inset). The MLBs are highly compound structures composed of “onion like” membrane stacks and linear membrane sheets (Figure 4C–E and Supplementary Figure S3, white arrows). These occupy a large volume of the organelle, segregating it into spaces occupied by newly formed virus particles (Figure 4 and Supplementary Figure S3, and Videos S1 and S2). The MLBs were also seen in non-infected cells (Supplementary Figure S4, and Video S3), suggesting these sites are not de novo generated as a consequence of VSV-S infection, but rather are exploited by the virus for its assembly. The VF contains virus particles with different morphologies that might represent various maturation stages of the particles. A portion of the virus particles contain electron dense material (red arrows in Figure 4C,F and Supplementary Figure S3, panel D), while others exhibit empty shells (green and purple arrows in Figure 4D,F and Supplementary Figure S3). Previous TEM studies of wild type VSV infected cells revealed a canal-like structure inside the virus particles [30]. In the current study, we observed a canal-like structure that occupies part of the volume of the particles. The nature of the canal is not known, but might represent defective particles or immature virus particles as was suggested before for wild type VSV [30] (yellow arrows in Figure 4C,D and Supplementary Figure S3, panel D).

The TEM tomography results demonstrate that there is more than one VF-containing MLB per cell and that the MLBs are connected in space, generating a continuous, complex, organized network of MLBs and host membrane cisternae (Videos S1 and S2).

### 3.3. VSV-S Genome Replication Occurs in Discrete Foci in the Cytoplasm Where VSV-S Proteins Accumulate

**Figure 5:**
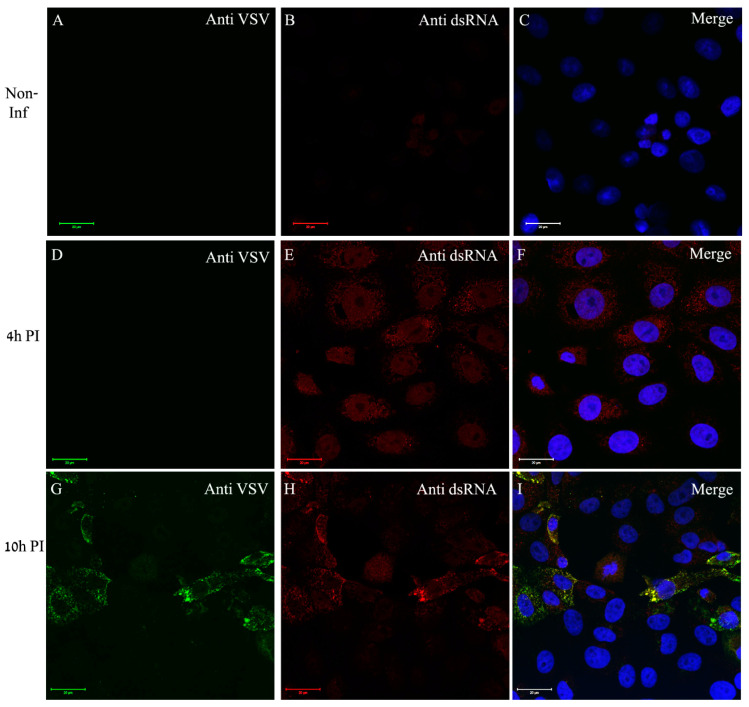
VSV-S genome replication occurs in discrete foci in the cytoplasm where VSV-S proteins accumulate. (**A**–**I**) Cells were either mock or VSV-S infected at the indicated time points and then processed by immuno-labeling with anti-VSV (**A**,**D**,**G**) and anti-dsRNA (**B**,**E**,**H**) antibodies. (**A**–**C**) Non-infected cells did not show any labeling of VSV proteins or dsRNA. (**D**–**F**) A representative image of cells infected for 4 h exhibiting no VSV proteins labeling. Scarce labeling of dsRNA in the cytoplasm is noticed. (**G**–**I**) At 10 h post infection, part of the cells were positive both for VSV proteins and dsRNA at distinct sites in the cytoplasm. (**C**,**F**,**I**) Merge channels of dsRNA (red), VSV proteins (green) and DAPI (blue) for nuclei staining. Scale bars are 20 µm. Objective lens X63.

In order to determine the locations of genome replication and viral proteins’ accumulation, we used immuno-labeling of VSV-S infected cells. dsRNA antibody was used to label dsRNA intermediates, a marker for RNA replication of both (+) and (−) RNA viruses, as was established in various RNA viruses including VSV [31]. Low labeling of dsRNA was noticed at 4 h PI in part of the cells but no viral proteins labeling was detected. However, at 10 h PI, co-localization of both viral proteins and dsRNA was seen in distinct sites in the cytoplasm (Figure 5).

**Figure 6:**
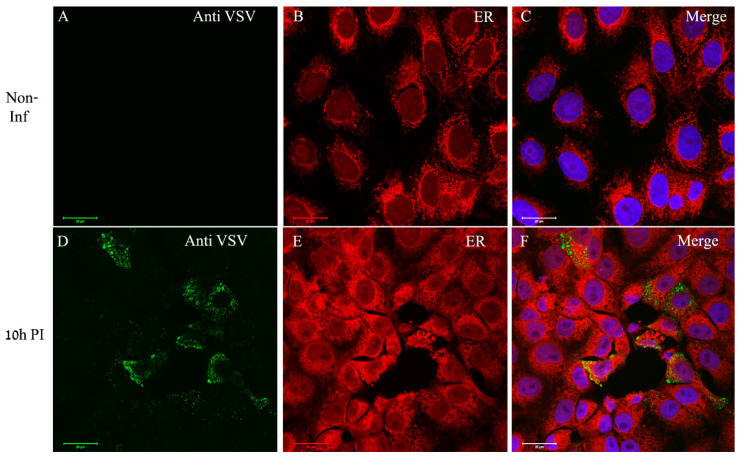
VSV-S proteins accumulate at distinct sites in the cytoplasm where ER is absent. (**A**–**F**) Cells were either mock or VSV-S infected for 10 h and then processed for immuno-labeling with anti-VSV antibodies (**A**,**D**) or stained for ER detection (**B**,**E**). (**A**–**C**) In non-infected cells the ER (red) accumulates around the nuclei. (**D**–**F**) At 10 h PI, VSV-S proteins (green) accumulate in regions where ER is absent. (**C**,**F**) Merge channels of both ER, VSV proteins and DAPI (blue) for nuclei staining. Scale bars are 20 µm. Objective lens X63.

In order to determine whether ER is recruited to viral proteins accumulation sites, the cells were dual labeled for ER and viral proteins. As can be seen, in infected cells the ER (red color) surrounds distinct punctuates in the cytoplasm (green) representing the viral proteins, but with no co-localization (Figure 6D–F). The overall immuno-labeling and ET results imply that the sites of viral genome replication, as well as viral proteins accumulation, most probably represent the VF, which are the sites for virus assembly, and are surrounded by ER membrane cisternae.

### 3.4. VSV-S Particles Are Found in Cytoplasmic Vacuoles Connected to Multi Lamellar Bodies Containing Viral Factories

**Figure 7:**
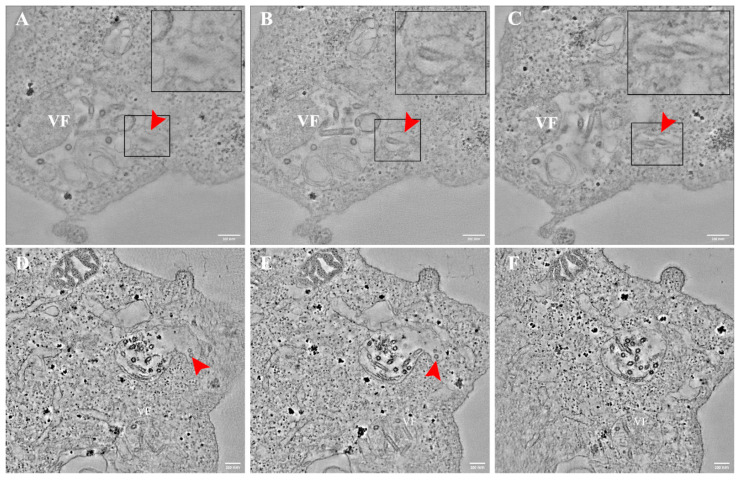
VSV-S particles are found in vacuoles after release from virus factories in multi lamellar bodies (MLB). (**A**–**F**) Sequential tomographic slices of VSV-S infected cells at 16 h (**A**–**C**) and 24 h post infection (PI) (**D**–**F**). Red arrows point toward several VSV-S particles inside a vacuole connected to MLB containing VF. Insets are high magnifications of delineated areas. Tomographic slices thicknesses are 13 nm (**A**–**C**) 4.3 nm (**D**–**F**). Scale bars are 200 nm.

Frequently, VSV-S particles were seen in vacuoles in the cytoplasm. By using ET, we were able to detect several vacuoles containing virus particles connected to the MLB containing VF (Figure 7 red arrows and Videos S4 and S5). It seems probable that these viruses are at the process of release from the MLB containing VF to a vacuole in the cytoplasm.

**Figure 8:**
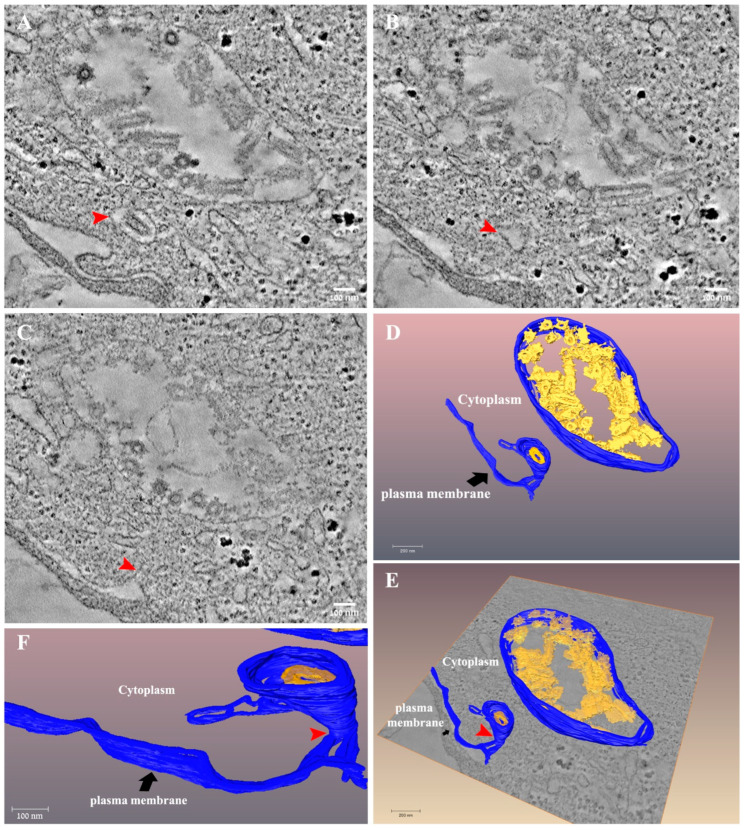
Release of VSV-S particles occurs after fusion of the vacuole membrane and the plasma membrane. (**A**–**C**) Sequential tomographic slices from a tomogram of 24 h post infection VSV-S infected cell. Red arrow points toward a VSV-S particle in a membrane vacuole (**A**). As the volume changes the vacuole becomes narrower ((**B**), red arrow) and at a certain point in the volume, is connected to a protrusion in the plasma membrane ((**C**), red arrow). (**D**–**F**) 3D surface rendering of the tomogram in panel (**A**). A large vacuole is depicted (blue color) with numerous virus particles decorated with probable spike proteins (yellow). A vacuole containing an individual virus particle is located nearby and is connected to the plasma membrane (blue) through a narrow membrane neck (red arrow). Tomographic slice thicknesses are 7 nm. Scale bars in panels (**A**–**C**) are 100 nm.

The infection cycle ends with the release of the newly formed virus particles to the extracellular space after fusion of the vacuole membrane with the plasma membrane (Figure 8 and Video S6). As shown, a large vacuole containing numerous virus particles decorated with “flask”-like projections resembling those of SARS-CoV-2 [32], most probably spike proteins, is found close to the plasma membrane. A single virus particle is seen in a neighboring vacuole (Figure 8A, red arrow), connected through a narrow membrane neck (red arrows in panels C, E, F) to the plasma membrane. This tomogram illustrates the budding process of newly formed VSV-S particles.

## 4. Discussion

The current study summarizes a thorough morphological study of various aspects of the infection cycle of VSV-S in Vero E6 cells. Initial events in the infection cycle were observed at 3–4 h PI, when viral RNA is replicated and accumulates in infected cells as infection progresses. This was evident by both RT real-time PCR and immuno-fluorescence labeling of dsRNA in VSV-S infected cells.

By using VSV serum antibodies and immuno-TEM labeling of VSV particles (Supplementary Figure S1), intense labeling was seen on the surface of wild type VSV, whereas in deformed VSV-S particles, released material from nucleocapsids was labeled, indicating that rabbit serum used for immuno-labeling assays contains antibodies against internal VSV-S antigens, but not spike protein. By using these antibodies in immuno-fluorescence assays, we observed that viral proteins accumulate at specific sites in the cytoplasm, where dsRNA localize but ER is absent. These results imply that dsRNA and VSV protein accumulation sites represent VF, said VF are sites where viral RNA and proteins accumulate in de novo synthesized virus particles. This conjecture is also supported by TEM tomography studies, showing VF containing various virus morphologies surrounded by membrane cisternae. Unfortunately, our attempts to specifically label VSV-S antigens by using rabbit serum antibodies (and also serum antibodies against Spike protein) on thin TEM sections of infected cells were unsuccessful. Previous studies on wild type-VSV and rabies infected cells showed that virus infection induces formation of cytoplasmatic inclusions where RNA synthesis co-localizes with viral proteins (such as N, L and P) [12,14,15,17,33]. ER membranes were seen in the vicinity of viral IBs, but not completely surrounding them. Although viral inclusions were devoid of host membranes at early stages of infections, double membrane cisternae (most probably ER) were seen adjacent to IBs at late stages of infection, and even completely surrounding them, concomitant with the budding of new particles from IBs [11]. After assembly, nucleocapsids are transported to the plasma membrane where glycoprotein (G protein) is assembled on their surface [33,34].

In the current study, we examined the differences between wild type VSV and VSV-S infected Vero E6, as we wanted to evaluate the effect of native G protein substitution with the spike protein. Thin section TEM analysis of wild type VSV and VSV-S infected Vero E6 cells showed that virus particles reside in cytoplasmatic vacuoles or at the outer leaflet of the plasma membrane (Figure 2 and Supplementary Figures S2). Similar results were obtained in a previous ultrastructural study conducted on wild VSV (Indiana strain) [30]. Nevertheless, no membrane-less, sphere-like inclusions were seen in their study and in our study as was shown by others [12]. The difference might be related to the following reasons: 1. Cell lines used in our study. 2. Unless directly labeled, inclusion bodies are difficult to recognize at early stages of infection, when virus particles and host membranes are yet to be seen related to them [11,14] 3. The structure of inclusion bodies changes over time. Indeed, inclusion bodies are dynamic structures, i.e., they change their morphology over the infection process, from spherical inclusions at early stages of infection to amorphous-like structures at late infection time points, when host membranes accumulate around them concomitant with the appearance of newly assembled nucleocapsids [11,14]. As, in the current study, we focus on late stages of infection, when viruses are produced in infected cells, it is easier to distinguish the VF.

Another major difference in the infection process of VSV-S compared to wild type VSV is that spike-like projections were seen on the surface of VSV-S located in cytoplasmatic vacuoles before fusion with the plasma membrane (Figure 8 and [32]), whereas in wild type VSV, “G” protein was shown to be assembled at budding sites located at specific microdomains [34]. It is tempting to speculate that substitution of native G and spike proteins change the cellular assembly location of these proteins on the surface of virus particles, but this has to be directly examined by specific labeling.

As mentioned above, VSV-S assembles in MLB. We were not able to determine the essence of these structures (whether these structures associate with autophagy vacuoles, lysosomes, or other cellular organelles), but we noticed that these are common in non-infected cells. Although the nature and the role of MLB in the infection process is not known, similar structures were previously observed in SARS-CoV infected Vero E6 cells [35]. These structures were associated with the formation of virus particles.

Other RNA viruses use diverse membrane-related cellular organelles to establish infection. Mitochondria, ER, Golgi, ER-Golgi intermediate compartments (ERGIC), or endosomes and lysosomes are used by different viruses (from various families) for the accumulation of viral factors needed for genome replication and virus assembly [4]. ER membrane re-organization or generation of novel membranous structures (originating from ER or Golgi membranes) are occasionally observed, for instance, after infection of cells with Flavivirus, Coronavirus or Bunyavirus [3,4,8,9,36]. Although the exact role of these membranes in infection is not known, we assume that ER is needed for viral protein translation, supporting membrane constituents of the MLB or protecting viral components from cellular RNA degrading enzymes.

As ET is limited to 200–300 nm section thickness, a whole MLB containing VF cannot be visualized in 3D. In order to compensate for this limitation, we performed 30 tomographic sessions of VSV-S infected cells at time points where VF are generated. Statistically, we assume that we have imaged sufficient orientation of the VF and thus gained adequate structural information.

Although in some tomograms it seems that the spike protein decorates part of the particles in the VF (for instance, yellow arrowsss in Figure 4C,D), attempts to specifically label it were unsuccessful.

**Figure 9:**
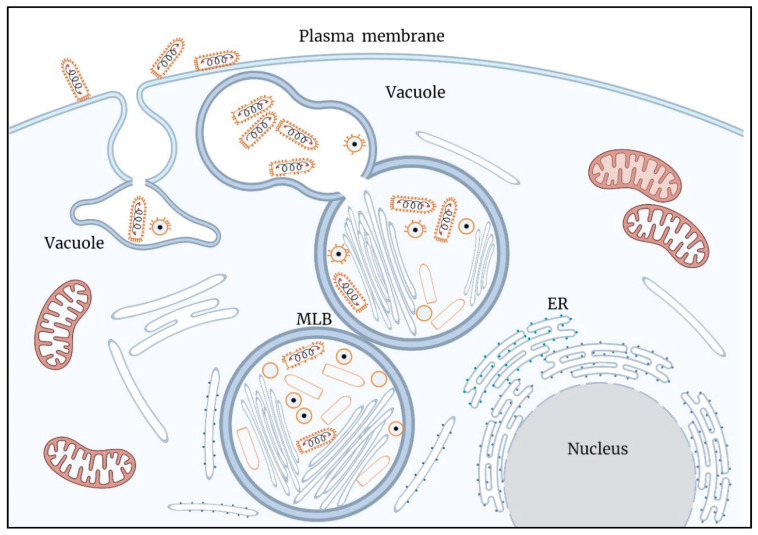
A model illustrating key processes in the infection cycle of VSV-S. Assembly and genome packaging of VSV-S particles is carried out in viral factories (VF) generated in multi lamellar bodies (MLB) located in the cytoplasm. Host ER membranes surround the MLB. Newly formed virus particles decorated by spike proteins are found in a vacuole connected to the MLB. Egress of VSV-S is accompanied by the fusion of the vacuole membrane and plasma membrane.

A model summarizing the results of this work is illustrated in Figure 9.

In conclusion, the current study describes detailed 3D imaging of VSV-S infection cycle. The infection shares common features of typical RNA viruses and may shed light on key processes in the infection cycle of wild type-VSV.

## Data Availability

Not applicable.

## Supplementary Materials

The following supporting information can be downloaded at: https://www.mdpi.com/article/10.3390/v14122828/s1, Figure S1: Immuno-TEM for detection of binding ability of VSV serum antibodies’ to VSV-S. Figure S2: Thin section TEM analysis of wild-type VSV and VSV-S infected Vero E6 cells. Figure S3: The viral factories of VSV-S are part of a complex network composed of host membrane cisternae and multi lamellar bodies. Figure S4: Electron tomography analysis of multi lamellar bodies in non-infected cells. Video S1: VSV-S viral factories are part of a complex network composed of cellular membranes and multi lamellar bodies. Video S2: The MLB containing VF generate a complex network web. Video S3: MLBs are native structures found in non-infected cells. Video S4: VSV-S particles are found in cytoplasmic vacuoles connected to virus factories in MLBs. Video S5: VSV-S particles are found in cytoplasmic vacuoles connected to virus factories. Video S6: Release of VSV-S particles occurs after fusion of the vacuole membrane and the plasma membrane.
