# Supplementary material for: Insights from the Infection Cycle of VSV-ΔG-Spike Virus
Source: Viruses. 2022 Dec 19;14(12):2828. doi: 10.3390/v14122828 (PMC9788095; doi:10.3390/v14122828)
Supplement: Supplementary file 1 [file viruses-14-02828-s001.zip › viruses-1816129-supplementary/viruses-1816129-supplementary.pdf]

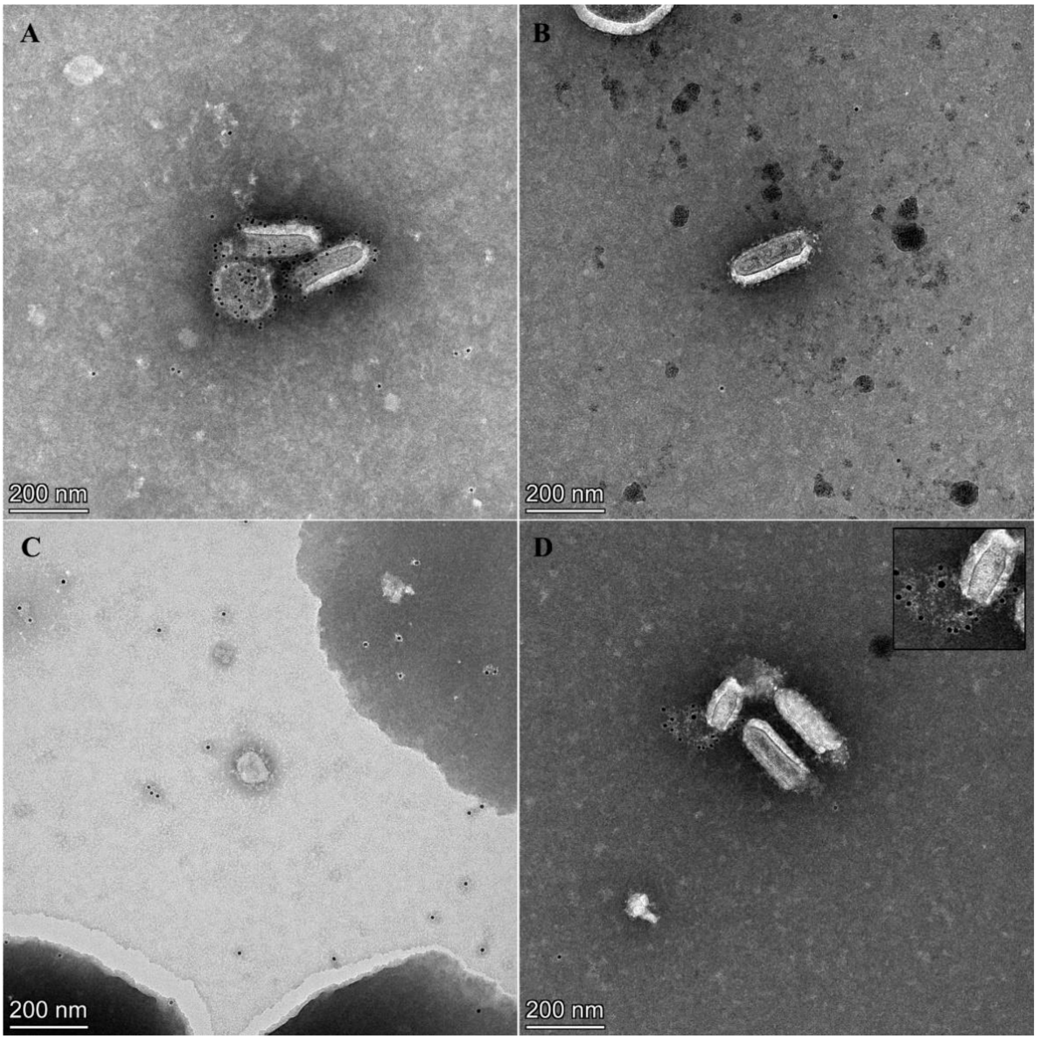

Figure S1. Immuno-TEM analysis of rabbit serum antibodies binding ability to VSV-S. **A–D.** TEM images of negatively stained and immunolabeled virus particles of wild-type VSV (**A**), VSV-S (**B**, **D**) and SARS-CoV-2 as negative control (**C**). (**A)** Wild type VSV particles exhibit pronounced labeling of their surface, most probably G protein. (**B)** The Spike proteins are clearly seen covering the surface of VSV-S without any labeling. (**C)** SARS-CoV-2 particles were used as a negative control indicating that the antibodies don't bind Spike proteins. (**D)** Labeling of antigens was seen in disrupted or partial disrupted VSV-S particles. Inset in panel **D** is high magnification of delineated area, showing partially disrupted particle with released material labeled with rabbit serum antibodies. The results suggest that rabbit serum contains antibodies against internal proteins of VSV-S as well as for surface proteins of VSV.

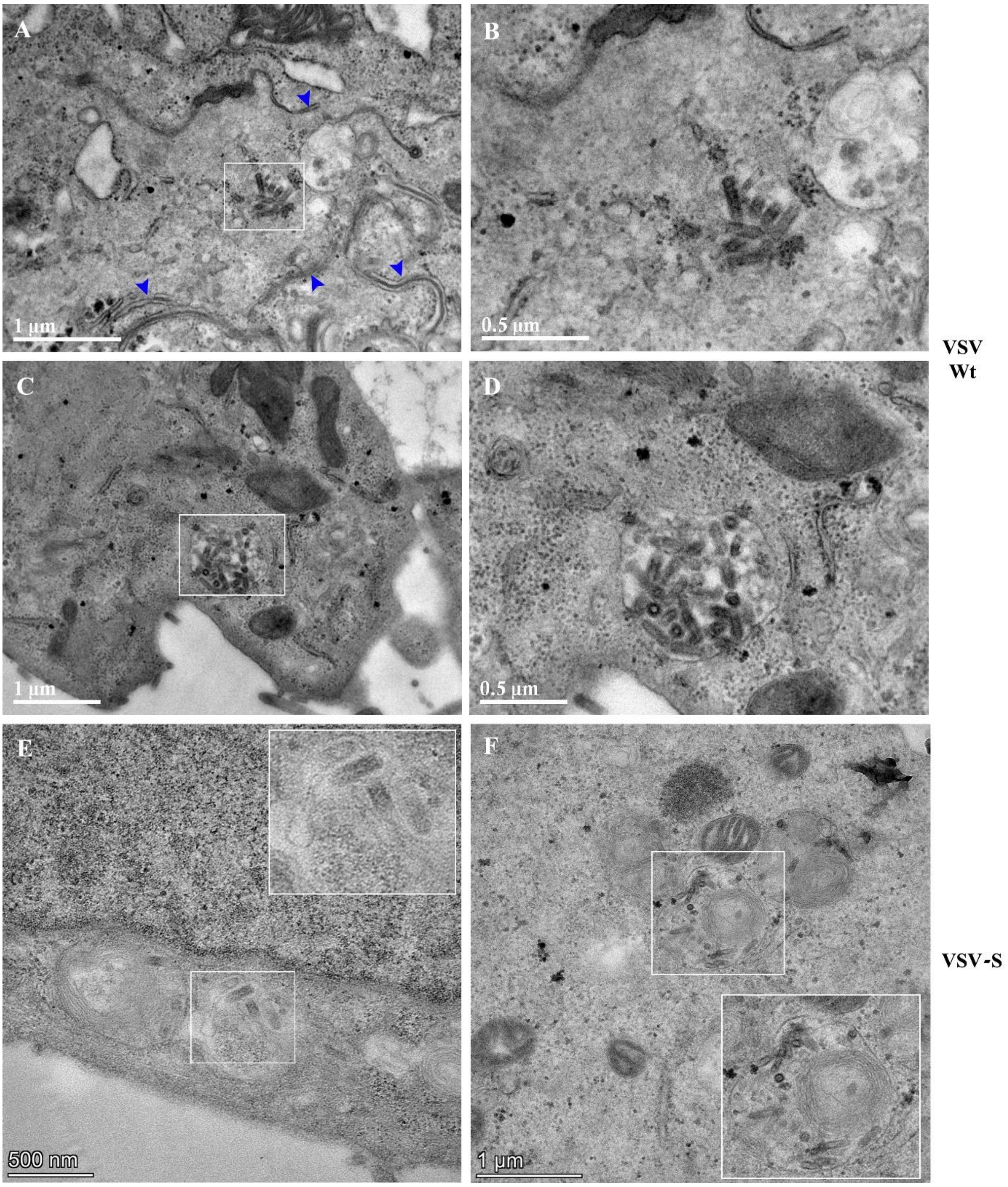

Figure S2. Thin section TEM analysis of wild type-VSV and VSV-S infected Vero E6 cells. (**A–D**). Wild type-VSV infected Vero E6 cells. (**A**) Low magnification view of an infected cell, showing virus particles with different morphologies in cytoplasmic sites (white inset) devoid of host organelles and ribosomes, and surrounded by host membranes (blue arrows). (**B**) High magnification view of the delineated area in panel A. Localized "bullet" shaped virus particles with electron dense material are seen in the cytoplasm. (**C**) Low magnification view of a different infected cells showing multiple virus particles inside a cytoplasmic vacuole. (**D**) High magnification view of the delineated area in panel C, showing VSV particles in transverse and longitudinal orientation inside a cytoplasmic vacuole. (**E, F**) VSV-S infected Vero E6 cells showing virus particles inside cytoplasmic vacuole (panel **E**) and multi lamellar bodies (panel **F**).

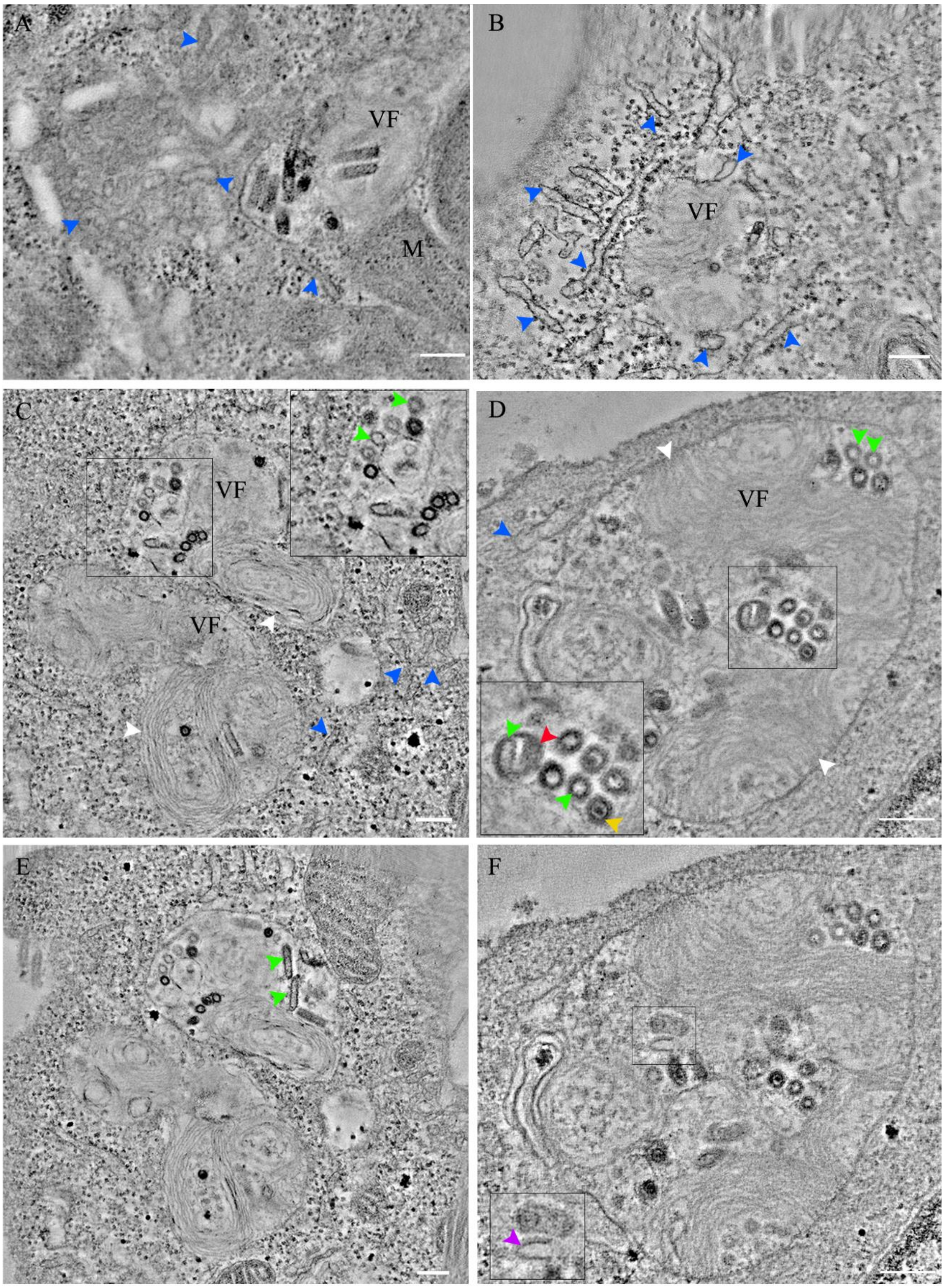

Figure S3. The viral factories (VF) of VSV-S are part of a complex network composed of host membrane cisternae and multi lamellar bodies (MLB). (**A–D)** Tomographic slices from four different tomograms of VSV-S infected cells at 10 hours (**A**), 16 hours (**B**, **C**) and 23 hours (**D**) post infection (PI). (**E, F)** These panels show sequential tomographic slices from the same tomograms in panels C and D, respectively. VSV-S VF are located in multi lamellar structures with adjacent host membrane cisternae (panels **A–D**, blue arrows). The VF consists of empty particles (panels **C–E**, green arrows) as well as viruses that already packed their genome (panel **D**, red arrow) or other morphologies exhibiting particles with partial density inside (Panel **D**, yellow arrow). Incomplete VSV-S particle that undergoes the process of assembly is labeled with purple arrow (inset shows high magnification of delineated area in panel **F**). White arrows point to "onion like" membrane stacks or linear

membrane sheets that are part of the MLB (panels **C**, **D**). VF - Viral factory, M - Mitochondria. Thickness of tomographic slices are 34 nm (**A**), 13 nm (**B**), 1.7 nm (**C**), 13 nm (**D**), 12 nm (**E**), 2 nm (**F**). Scale bars are 200 nm.

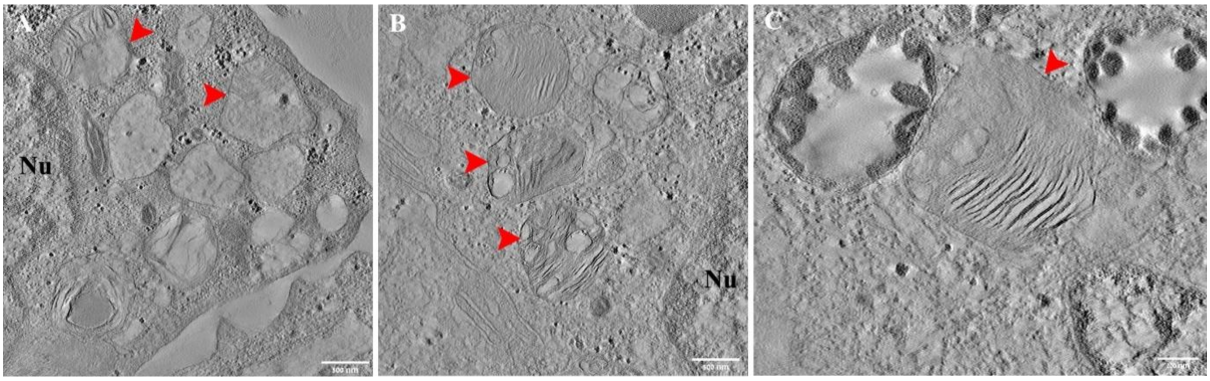

Figure S4. Electron tomography analysis of multi lamellar bodies in non-infected cells. (**A–C)** Tomographic slices from three different tomograms of non-infected cells showing several adjacent lamellar bodies (red arrows). No virus particles could be seen in the lamellar bodies. Scale bars are 500 nm (**A**, **B**) and 200 nm (**C**). Nu – Nucleus. Thickness of tomographic slices in panels A and B are 2.15 nm and panel **C** is 1.33 nm.

Video S1. VSV-S viral factories (VF) are part of a complex network composed of cellular membranes and multi lamellar bodies (MLB). This video shows a tomogram of a 16 hour post infection VSV-S infected cell. The VF is located in MLB which has interior compartments filled with newly formed virus particles. Notably, host membrane cisternae are seen at the periphery of the MLB.

Video S2. The multi lamellar bodies (MLB) containing viral factories (VF) generate a complex network. This video shows a tomogram of VSV-S infected cell for 16 hours post infection. The VF consist of empty particles as well as viruses that already packed their genome or at intermediate assembly stages. The VF containing MLB are inter-connected and generate a network.

Video S3. Multi lamellar bodies are native structures found in non-infected cells. This video shows a tomogram of a non-infected cell. The multi lamellar bodies are seen devoid of virus particles. This video illustrates that these structures are not induced by viral infection.

Video S4. VSV-S particles are found in cytoplasmic vacuoles connected to virus factories (VF) in multi lamellar bodies. This video shows a tomogram of VSV-S infected cell for 16 hours post infection. A vacuole containing two virus particles is seen connected to the VF.

Video S5. VSV-S particles are found in cytoplasmic vacuoles connected to virus factories (VF). This video shows a tomogram of VSV-S infected cell for 24 hours post infection. A VF containing numerous virus particles that are in part coated with a probable spike protein is observed. A vacuole connected to the VF and containing two additional virus particles, is observed at a certain point in the volume of the tomogram.

Video S6. Release of VSV-S particles occurs after fusion of the vacuole membrane and the plasma membrane. This video shows a tomogram of VSV-S infected cell for 24 hours post infection. A virus containing vacuole is connected through a narrow neck to the plasma membrane. In addition, a larger vacuole containing spike decorated VSV-S particles waiting for release is seen nearby.
